# Proportionate action requires a decision rule

**DOI:** 10.1016/j.lana.2026.101637

**Published:** 2026-09-09

**Authors:** Nilson N. Mendes Neto, Jessika M. Mendes

**Affiliations:** a Clinical Investigation, Harvard Medical School, Boston, MA, USA; b Pain Psychiatry Fellowship, Natal, RN, Brazil; c Teaching Pain Clinic, Universidade Potiguar, Natal, RN, Brazil

The recent Editorial on youth suicide prevention in the Americas argues that emerging evidence is no reason to postpone proportionate action.^1^ We agree. Proportionality, however, remains to be specified as a decision rule. This matters because youth suicide exemplifies an evidence paradox: the populations at greatest risk are often those in whom definitive trials are hardest to conduct.^2^ The unresolved question is therefore what evidentiary threshold should justify action when both intervention and inaction can cause harm.

Universal suicide-risk screening illustrates the difficulty. The US Preventive Services Task Force concludes that evidence is insufficient to determine the balance of benefits and harms of screening children and adolescents for suicide risk.^3^ In settings with limited referral capacity, false positives and downstream demand can themselves generate opportunity costs. Conversely, delaying potentially beneficial interventions while awaiting definitive evidence can also carry substantial costs. A proportionate threshold should therefore vary with the asymmetry of expected harms: it should be lower when the consequences of inaction are substantial and an intervention is low-burden, reversible, and monitorable, and higher when an intervention is coercive, irreversible, or system-intensive. The capacity to learn after implementation does not determine whether action is justified, but it affects how much uncertainty can reasonably be tolerated.

This decision rule also changes the role of surveillance. The Editorial calls for stronger and more disaggregated data across highly heterogeneous populations and settings.^1^ Uneven adoption of policies affecting digital environments, schools, and primary care can also create opportunities for natural experiments. When intervention timing and exposure are documented prospectively and linked to appropriate outcome data and comparison groups, quasi-experimental designs can strengthen causal inference where randomisation is impractical.^4^

Evaluability, however, cannot become a prerequisite for action. Such a requirement would systematically disadvantage settings with weaker information systems, which may also carry substantial unmet need. Building the capacity to detect effects and learn from implementation should therefore be part of implementation itself rather than a condition imposed before it begins.

Proportionate action is not simply action despite uncertainty. It is action calibrated to the consequences of being wrong and, where feasible, designed to make subsequent decisions less uncertain.

## Declaration of interests

The authors declare no competing interests.
